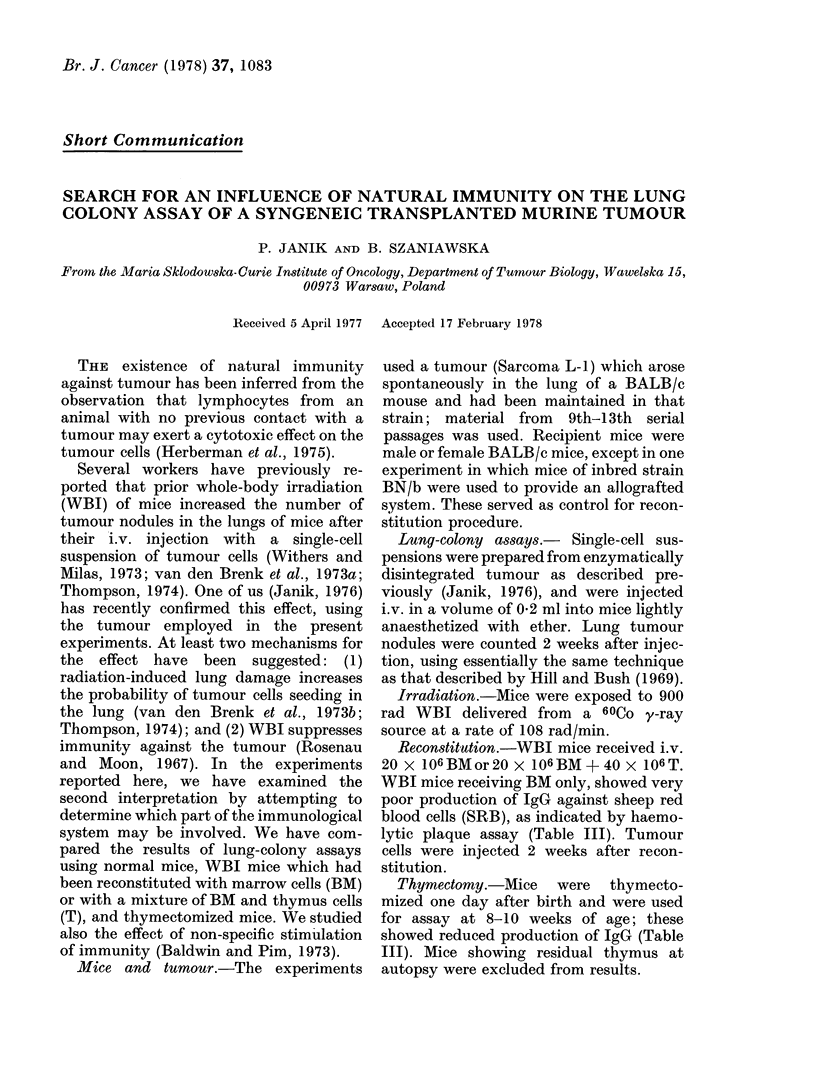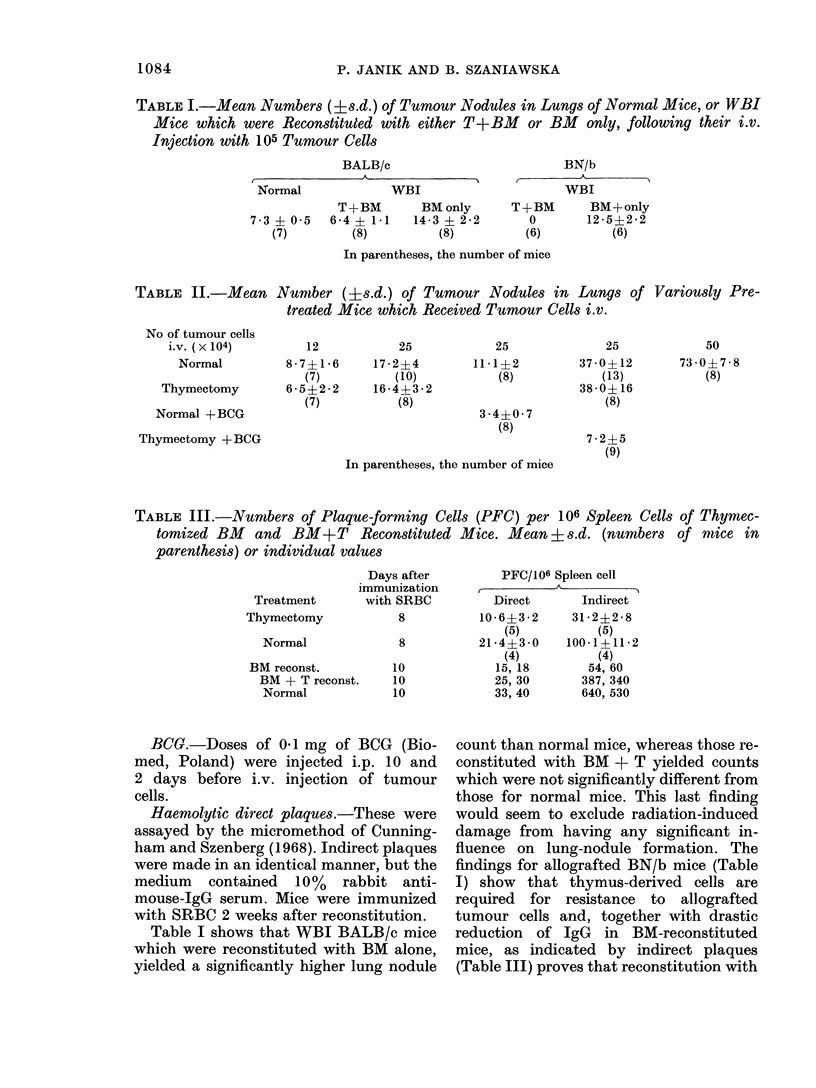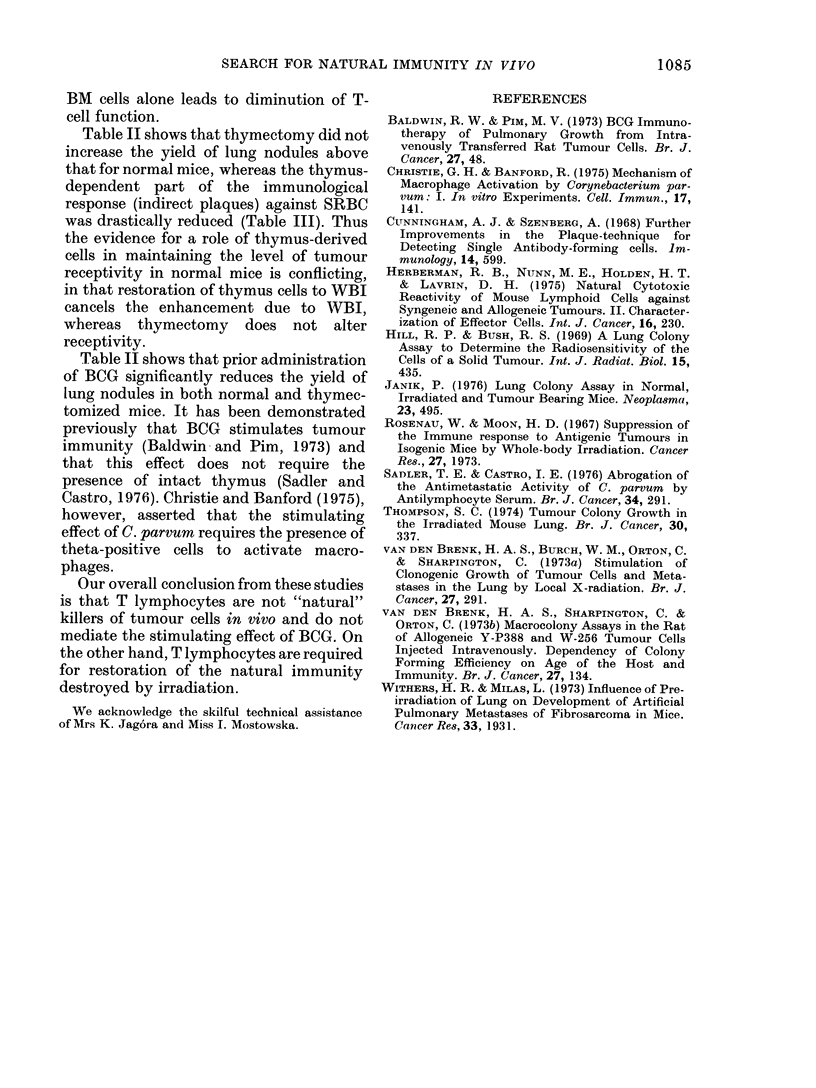# Search for an influence of natural immunity on the lung colony assay of a syngeneic transplanted murine tumour.

**DOI:** 10.1038/bjc.1978.157

**Published:** 1978-06

**Authors:** P. Janik, B. Szaniawska


Br. J. Cancer (1978) 37, 1083

### Short Communication

### SEARCH FOR AN INFLUENCE OF NATURAL IMMUNITY ON THE LUNG COLONY ASSAY OF A SYNGENEIC TRANSPLANTED MURINE TUMOUR

P. JANIK AND B. SZANIAWSKA

*From the Maria Sklodowska-Curie Institute of Oncology, Department of Tumour Biology, Wawelska 15, 00973 Warsaw, Poland*

Received 5 April 1977 Accepted 17 February 1978

THE existence of natural immunity against tumour has been inferred from the observation that lymphocytes from an animal with no previous contact with a tumour may exert a cytotoxic effect on the tumour cells (Herberman *et al.*, 1975).

Several workers have previously reported that prior whole-body irradiation (WBI) of mice increased the number of tumour nodules in the lungs of mice after their i.v. injection with a single-cell suspension of tumour cells (Withers and Milas, 1973; van den Brenk *et al.*, 1973*a*; Thompson, 1974). One of us (Janik, 1976) has recently confirmed this effect, using the tumour employed in the present experiments. At least two mechanisms for the effect have been suggested: (1) radiation-induced lung damage increases the probability of tumour cells seeding in the lung (van den Brenk *et al.*, 1973*b*; Thompson, 1974); and (2) WBI suppresses immunity against the tumour (Rosenau and Moon, 1967). In the experiments reported here, we have examined the second interpretation by attempting to determine which part of the immunological system may be involved. We have compared the results of lung-colony assays using normal mice, WBI mice which had been reconstituted with marrow cells (BM) or with a mixture of BM and thymus cells (T), and thymectomized mice. We studied also the effect of non-specific stimulation of immunity (Baldwin and Pim, 1973).

*Mice and tumour.*—The experiments used a tumour (Sarcoma L-1) which arose spontaneously in the lung of a BALB/c mouse and had been maintained in that strain; material from 9th–13th serial passages was used. Recipient mice were male or female BALB/c mice, except in one experiment in which mice of inbred strain BN/b were used to provide an allografted system. These served as control for reconstitution procedure.

*Lung-colony assays.*— Single-cell suspensions were prepared from enzymatically disintegrated tumour as described previously (Janik, 1976), and were injected i.v. in a volume of 0·2 ml into mice lightly anaesthetized with ether. Lung tumour nodules were counted 2 weeks after injection, using essentially the same technique as that described by Hill and Bush (1969).

*Irradiation.*—Mice were exposed to 900 rad WBI delivered from a $^{60}$Co $\gamma$-ray source at a rate of 108 rad/min.

*Reconstitution.*—WBI mice received i.v. $20 \times 10^6$ BM or $20 \times 10^6$ BM + $40 \times 10^6$ T. WBI mice receiving BM only, showed very poor production of IgG against sheep red blood cells (SRB), as indicated by haemolytic plaque assay (Table III). Tumour cells were injected 2 weeks after reconstitution.

*Thymectomy.*—Mice were thymectomized one day after birth and were used for assay at 8–10 weeks of age; these showed reduced production of IgG (Table III). Mice showing residual thymus at autopsy were excluded from results.

TABLE I.—*Mean Numbers (±s.d.) of Tumour Nodules in Lungs of Normal Mice, or WBI Mice which were Reconstituted with either T+BM or BM only, following their i.v. Injection with* $10^5$ *Tumour Cells*

| BALB/c | | | BN/b | |
|---|---|---|---|---|
| Normal | WBI | | WBI | |
| | T+BM | BM only | T+BM | BM+only |
| 7·3 ± 0·5 (7) | 6·4 ± 1·1 (8) | 14·3 ± 2·2 (8) | 0 (6) | 12·5±2·2 (6) |

In parentheses, the number of mice

TABLE II.—*Mean Number (±s.d.) of Tumour Nodules in Lungs of Variously Pretreated Mice which Received Tumour Cells i.v.*

| No of tumour cells i.v. ($\times 10^4$) | 12 | 25 | 25 | 25 | 50 |
|---|---|---|---|---|---|
| Normal | 8·7±1·6 (7) | 17·2±4 (10) | 11·1±2 (8) | 37·0±12 (13) | 73·0±7·8 (8) |
| Thymectomy | 6·5±2·2 (7) | 16·4±3·2 (8) | | 38·0±16 (8) | |
| Normal +BCG | | | 3·4±0·7 (8) | | |
| Thymectomy +BCG | | | | 7·2±5 (9) | |

In parentheses, the number of mice

TABLE III.—*Numbers of Plaque-forming Cells (PFC) per* $10^6$ *Spleen Cells of Thymectomized BM and BM+T Reconstituted Mice. Mean±s.d. (numbers of mice in parenthesis) or individual values*

| Treatment | Days after immunization with SRBC | PFC/$10^6$ Spleen cell Direct | Indirect |
|---|---|---|---|
| Thymectomy | 8 | 10·6±3·2 (5) | 31·2±2·8 (5) |
| Normal | 8 | 21·4±3·0 (4) | 100·1±11·2 (4) |
| BM reconst. | 10 | 15, 18 | 54, 60 |
| BM + T reconst. | 10 | 25, 30 | 387, 340 |
| Normal | 10 | 33, 40 | 640, 530 |

*BCG.*—Doses of 0·1 mg of BCG (Biomed, Poland) were injected i.p. 10 and 2 days before i.v. injection of tumour cells.

*Haemolytic direct plaques.*—These were assayed by the micromethod of Cunningham and Szenberg (1968). Indirect plaques were made in an identical manner, but the medium contained 10% rabbit anti-mouse-IgG serum. Mice were immunized with SRBC 2 weeks after reconstitution.

Table I shows that WBI BALB/c mice which were reconstituted with BM alone, yielded a significantly higher lung nodule count than normal mice, whereas those reconstituted with BM + T yielded counts which were not significantly different from those for normal mice. This last finding would seem to exclude radiation-induced damage from having any significant influence on lung-nodule formation. The findings for allografted BN/b mice (Table I) show that thymus-derived cells are required for resistance to allografted tumour cells and, together with drastic reduction of IgG in BM-reconstituted mice, as indicated by indirect plaques (Table III) proves that reconstitution with

BM cells alone leads to diminution of T-cell function.

Table II shows that thymectomy did not increase the yield of lung nodules above that for normal mice, whereas the thymus-dependent part of the immunological response (indirect plaques) against SRBC was drastically reduced (Table III). Thus the evidence for a role of thymus-derived cells in maintaining the level of tumour receptivity in normal mice is conflicting, in that restoration of thymus cells to WBI cancels the enhancement due to WBI, whereas thymectomy does not alter receptivity.

Table II shows that prior administration of BCG significantly reduces the yield of lung nodules in both normal and thymectomized mice. It has been demonstrated previously that BCG stimulates tumour immunity (Baldwin and Pim, 1973) and that this effect does not require the presence of intact thymus (Sadler and Castro, 1976). Christie and Banford (1975), however, asserted that the stimulating effect of *C. parvum* requires the presence of theta-positive cells to activate macrophages.

Our overall conclusion from these studies is that T lymphocytes are not "natural" killers of tumour cells *in vivo* and do not mediate the stimulating effect of BCG. On the other hand, T lymphocytes are required for restoration of the natural immunity destroyed by irradiation.

We acknowledge the skilful technical assistance of Mrs K. Jagóra and Miss I. Mostowska.

### REFERENCES

BALDWIN, R. W. & PIM, M. V. (1973) BCG Immunotherapy of Pulmonary Growth from Intravenously Transferred Rat Tumour Cells. *Br. J. Cancer*, **27**, 48.

CHRISTIE, G. H. & BANFORD, R. (1975) Mechanism of Macrophage Activation by *Corynebacterium parvum*: I. *In vitro* Experiments. *Cell. Immun.*, **17**, 141.

CUNNINGHAM, A. J. & SZENBERG, A. (1968) Further Improvements in the Plaque-technique for Detecting Single Antibody-forming cells. *Immunology*, **14**, 599.

HERBERMAN, R. B., NUNN, M. E., HOLDEN, H. T. & LAVRIN, D. H. (1975) Natural Cytotoxic Reactivity of Mouse Lymphoid Cells against Syngeneic and Allogeneic Tumours. II. Characterization of Effector Cells. *Int. J. Cancer*, **16**, 230.

HILL, R. P. & BUSH, R. S. (1969) A Lung Colony Assay to Determine the Radiosensitivity of the Cells of a Solid Tumour. *Int. J. Radiat. Biol.* **15**, 435.

JANIK, P. (1976) Lung Colony Assay in Normal, Irradiated and Tumour Bearing Mice. *Neoplasma*, **23**, 495.

ROSENAU, W. & MOON, H. D. (1967) Suppression of the Immune response to Antigenic Tumours in Isogenic Mice by Whole-body Irradiation. *Cancer Res.*, **27**, 1973.

SADLER, T. E. & CASTRO, I. E. (1976) Abrogation of the Antimetastatic Activity of *C. parvum* by Antilymphocyte Serum. *Br. J. Cancer*, **34**, 291.

THOMPSON, S. C. (1974) Tumour Colony Growth in the Irradiated Mouse Lung. *Br. J. Cancer*, **30**, 337.

VAN DEN BRENK, H. A. S., BURCH, W. M., ORTON, C. & SHARPINGTON, C. (1973*a*) Stimulation of Clonogenic Growth of Tumour Cells and Metastases in the Lung by Local X-radiation. *Br. J. Cancer*, **27**, 291.

VAN DEN BRENK, H. A. S., SHARPINGTON, C. & ORTON, C. (1973*b*) Macrocolony Assays in the Rat of Allogeneic Y-P388 and W-256 Tumour Cells Injected Intravenously. Dependency of Colony Forming Efficiency on Age of the Host and Immunity. *Br. J. Cancer*, **27**, 134.

WITHERS, H. R. & MILAS, L. (1973) Influence of Preirradiation of Lung on Development of Artificial Pulmonary Metastases of Fibrosarcoma in Mice. *Cancer Res*, **33**, 1931.